# Improving the safety of systemic viral gene therapy

**DOI:** 10.1172/JCI177078

**Published:** 2024-01-02

**Authors:** Elizabeth M. McNally

Systemic treatment with adeno-associated virus (AAV) gene replacement therapy has seen gains with FDA approval for onasemnogene abeparvovec (trade name Zolgensma) in 2019 to treat spinal muscular atrophy (SMA) and approval for delandistrogne (trade name Elevidys) in 2023 to treat Duchenne muscular dystrophy (DMD) (1). Additional ongoing clinical trials are evaluating the efficacy and safety of systemic AAV gene therapy for multiple rare genetic conditions. Experience in systemic AAV dosing is providing a wealth of information about side effects from gene therapy, especially the immune response to AAV capsid and the expressed products; however, much of the information gleaned remains uncurated and dispersed among institutions. In this issue of the *JCI*, Salabarria and colleagues report on their study with alternative immunosuppression strategies in human patients receiving approved or experimental AAV9 gene therapy (2). Notably, the authors cataloged data from 38 children who received several different gene therapy protocols and collated the information at a single center (2).

Systemic AAV gene therapy requires substantial doses, typically 1 × 10^13^ to 2 × 10^14^ vector genomes per kg, which trigger both innate and humoral responses (3). Thrombotic microangiopathy (TMA), a potentially life-threatening condition, can occur relatively early after dosing and is characterized by thrombocytopenia, hemolytic anemia, and end-organ damage to the heart, lungs, kidneys, and other organs. In a nonrandomized series, Salabarria et al. (2) evaluated patients receiving AAV9 for multiple conditions, including SMA, GM1 gangliosidosis, DMD, and Danon disease. The patients were divided into two groups; one group received only corticosteroids, while the second group additionally received rituximab and sirolimus. After AAV dosing, Group 1 patients showed a spike in anti-capsid IgG and IgM antibodies, complement activation, and a reduction in platelets. In contrast, the Group 2 patients did not have the acute rise in anti-capsid IgG and IgM antibodies nor did they display the same degree of complement activation or platelet drop. The authors conclude that a conditioning regimen with rituximab and sirolimus can reduce the likelihood of TMA. By the nature of the study design, the groups were unequal in age and the specific gene therapy being delivered, so additional studies are needed to evaluate this conditioning regimen and others.

Lek et al. recently reported the death of a young adult with DMD who received AAV gene therapy to drive dystrophin upregulation using an inactivated Cas9-VP64 domain (4). Despite receiving rituximab and sirolimus, the patient died with cardiac and pulmonary failure approximately one week after receiving gene therapy. The ante- and postmortem studies documented thrombocytopenia, some complement activation, and a preexisting severe cardiomyopathy. The timing of death and molecular studies indicated immunity to the AAV capsid itself, rather than the gene editing machinery, as no expression of the gene editing machinery was detected.

The unique diversity of the human immune system and its reaction to high-dose AAV is poorly modeled in preclinical animal studies, so it is only through human AAV gene therapy trials that more can be learned. An array of immunomodulatory agents is available to manage outcomes to viral gene therapy, and immunomodulation regimens are often not precisely stipulated by the clinical trial design or the package label. Immunomodulatory management of viral gene therapy is not unlike organ transplant management. For organ transplantation, registries have been invaluable to record management and outcomes. Indeed, a recent report of immune responses in three different AAV gene therapy trials for DMD was able to identify a target antigen driving an immune response only because these trials shared common investigators (5). The work by Salabarria et al. (2) reminds us that registries for cataloging immune management and outcomes can improve safety and efficacy of AAV for all and should be considered for gene therapy.

## Version 1 01/02/2024

Electronic publication
